# Wavelet Frequency Separation Attention Network for Chest X-ray Image Super-Resolution

**DOI:** 10.3390/mi12111418

**Published:** 2021-11-18

**Authors:** Yue Yu, Kun She, Jinhua Liu

**Affiliations:** 1 School of Information and Software Engineering, University of Electronic Science and Technology of China, Chengdu 610054, China; thomasyyu@163.com (Y.Y.); kun@uestc.edu.cn (K.S.); 2 School of Mathematical and Computer Sciences, Shangrao Normal University, Shangrao 334001, China

**Keywords:** medical imaging, stationary wavelet transform, ghost module, attention mechanism

## Abstract

Medical imaging is widely used in medical diagnosis. The low-resolution image caused by high hardware cost and poor imaging technology leads to the loss of relevant features and even fine texture. Obtaining high-quality medical images plays an important role in disease diagnosis. A surge of deep learning approaches has recently demonstrated high-quality reconstruction for medical image super-resolution. In this work, we propose a light-weight wavelet frequency separation attention network for medical image super-resolution (WFSAN). WFSAN is designed with separated-path for wavelet sub-bands to predict the wavelet coefficients, considering that image data characteristics are different in the wavelet domain and spatial domain. In addition, different activation functions are selected to fit the coefficients. Inputs comprise approximate sub-bands and detail sub-bands of low-resolution wavelet coefficients. In the separated-path network, detail sub-bands, which have more sparsity, are trained to enhance high frequency information. An attention extension ghost block is designed to generate the features more efficiently. All results obtained from fusing layers are contracted to reconstruct the approximate and detail wavelet coefficients of the high-resolution image. In the end, the super-resolution results are generated by inverse wavelet transform. Experimental results show that WFSAN has competitive performance against state-of-the-art lightweight medical imaging methods in terms of quality and quantitative metrics.

## 1. Introduction

At present, medical images provide an important basis for disease diagnosis. Wavelet-based medical imaging has attracted much attention [1,2]. Generally speaking, conventional medical imaging systems typically include magnetic resonance imaging (MRI) [3], computed tomography (CT) [4], and positron emission computed tomography (PET-CT) [5]. MRI is more suitable for the detection of the brain and soft tissue, whereas CT is more often used for bone and chest. High resolution (HR) medical images provide richer details and better visual quality; they play an important role in experts’ diagnosis. However, due to the high cost of hardware equipment and the limitation of imaging technology in a specific situation, obtaining high-resolution medical images by super-resolution has been an important trend [6]. In addition, due to factors such as device configuration, limited scanning time, and body motion, these images with noise and lack of structural information often have low resolution (LR). In such scenarios, super-resolution is preferred by medical professionals to enhance medical images.

Super-resolution is a classical ill-posed inverse problem given the multiple approaches to reconstruct HR images. The medical image super-resolution is addressed by single image super-resolution (SISR), which refers to recovery of information of the corresponding HR image from a single LR input. The single image-based methods can be classified as: interpolation based [7,8], edge directed [9,10], sparsity based [11,12,13,14,15], and deep learning based [16,17,18,19]. Among these methods, sparse coding-based (SC) methods [11,12], as representative sparsity methods, are inspired by the research, where image patches can be represented as a sparse linear combination of elements with an appropriate over-complete dictionary selection. A sparse representation of each low-resolution patch, which is captured from the input image, and the sparse coefficients are used to generate the high-resolution patch. Finally, the high-resolution image is reconstructed by the output patches. Furthermore, the literature [14,15] exploits the structure of sparse and nonlocal self-similarity priors for recovering images. However, the sparse-based super resolution requires human experience to set the relevant parameters, thereby resulting in the loss of image detail information and overly smooth reconstruction findings [13].

Recently, deep learning approaches and neural network models have become more popular since Dong et al. [16] proposed the super-resolution convolutional neural network (SRCNN) model. Instead of learning the dictionaries directly, SRCNN learns an end-to-end mapping between low- and high-resolution images. This model conceptually consists of three parts, namely patch extraction and representation, nonlinear mapping, and reconstruction. With its three-layer convolutional network structure, SRCNN reconstructs its high-resolution image rapidly and maintains high quality at the same time. Thus, many modified SRCNN models have been proposed. Loy et al. [17] proposed a fast super-resolution convolutional neural network (FSRCNN) with improvements to accelerate the SRCNN model. This method adopts a deconvolution layer to compose the sample, while it utilizes the shrinking, mapping, and expanding layers to replace the nonlinear mapping layers. The smaller filer sizes and the deeper network structure also reduce the computational cost and improve the performance. Lim et al. [18] implemented an enhanced deep super-resolution network and a new multiscale deep super-resolution system, where batch normalization layers are removed in the network. Ledig et al. [19] presented a generative adversarial network for image super-resolution (SRGAN) with the generative adversarial nets(GAN) [20]. Wang et al. [21] proposed an enhanced SRGAN (ESRGAN) by introducing the residual-in-residual dense block without batch normalization to enhance the visual quality. As we know, the usage of deep residual learning (ResNet) [22] in very deep convolution networks (VDSR) increases the depth of the network to 20 layers to obtain higher accuracy and visual improvements. Tong et al. [23] proposed SRDenseNet by using dense connected convolutional networks [24]. It demonstrates that the combination of features at different levels improves the performance. Woo et al. [25] proposed a convolutional block attention model(CBAM), which obtained satisfied result. Furthermore, Hou et al. [26] adopts the alternative upscaled and downscaled layers in the generator with relativistic disciminator to capture the high-resolution image from extreme low-resolution image. Moreover, Zhang et al. [27] presented a fast medical super resolution (FMISR) method, which contributes to the mini-network and uses the sub-pixel convolution layer. Shi et al. [28] designed an efficient sub-pixel convolutional neural network model. These deep learning-based methods can be address the image super resolution problem and have achieved favorable results. However, most methods are aimed at conventional natural images. In particular, the above methods might produce undesired artefacts in HR images when performed on medical images.

The main purpose of our study is to design a lighter medical imaging super-resolution model, which is named WFSAN model. The WFSAN model integrates the sparseness of wavelet-based methods and the advantages of learning-based methods and provides an avenue to bridge the gap between wavelet-based and learning-based methods. Furthermore, our model has few parameters, has competitive parameters and visual effect, and performs favorably on LR images with different degradation settings, showing great potential for practical applications such as CT or MRI imaging. In this work, we address the problem of single medical image super resolution in wavelet domain. We anchor the focus on the data feature in different sub-bands to take advantage of the feature of wavelet domain. On the basis of the analyzed fact that the distribution of approximate frequency sub-band and detail frequency sub-band is different, a wavelet frequency separation network is adopted to enhance learning the features of each sub-band, thereby accelerating the convergence speed and improving the accuracy. The approximate frequency sub-bands represent average information, and detail frequency sub-bands include horizontal, vertical, and diagonal information. Consequently, the network is designed to obtain the sparse representation of these frequency sub-bands. The input tensor inside the high-frequency feature extraction path is divided into horizontal, vertical, and diagonal sub-bands. An attention ghost extension block with fewer parameters is designed to contain more information for each path. The features of all sub-bands are fused to reconstruct the predicted wavelet coefficients. Suitable activate functions are selected in each path of the feature extraction net and the reconstruction net.

The main contributions can be summarized as follows:

1 In the existing wavelet-based deep learning approaches, wavelet-based deep learning approaches, the first approach analyzes and utilizes the numeric features for each sub-bands in the wavelet domain and processes them separately; other methods mainly consider the different characteristics between spatial and wavelet domain.
2 Instead of learning the features of all sub-bands together, we propose a wavelet frequency separation network model to capture the features for each separated frequency sub-band and enhance the high-frequency feature. Attention ghost extension block is designed to obtain more information with fewer parameters.These features are fused by a designed attention fusing block to form the high-resolution image.
3 In this end-to-end network of multiple input and output channels in the wavelet domain, the sparsity and image structure information provided by low-frequency and high-frequency sub-bands of discrete wavelet transform are utilized, respectively.

## 2. Related Work

### 2.1. Wavelet-Based Image Super Resolution

In recent years, in order to take advantage of the sparsity and multiresolution of wavelet transform [29], a surge of approaches [30,31,32,33,34,35] with the wavelet technology have been proposed on image super resolution. Among these algorithms, [30,31,32,33] adopt the combination of the discrete wavelet transform and sparse representation instead of deep learning to obtain the HR image. Guo et al. [34] proposed DWSR as the first approach to predict high-resolution images in wavelet domain with a deep CNN network. The super-resolution problems are transformed into the prediction problem of wavelet coefficients with one-level discrete wavelet transform. The performance of the model is enhanced owing to the sparsity brought by the wavelet coefficients. A residual net is built by learning the residual coefficients between low resolution image and high resolution image. Huang et al. [35] implemented a wavelet-based CNN (Wavelet-SRNet) for multi-scale face super resolution. The one-level discrete wavelet transform is replaced by the wavelet packet decomposition. Skip connections exist in the embedding and wavelet predicting networks, and the reconstruction network comprises deconvolution layers. Wavelet prediction loss, texture loss, and full-image loss are used together to maintain training stability and prevent the degradation of texture details. The discrete wavelet transform combined with recursive Res-Net WTCCR [36] explored the possibilities of depicting images at different sub-bands. It replaces the low-frequency sub-band by LR image to gain more details. For medical imaging super resolution, Deeba et al. [37] proposed a wavelet-based enhanced medical image super resolution (WMSR) method, which adopts the combination of the one-level discrete stationary wavelet transform and a mini-gird network rather than the combination of the discrete wavelet transform and a convolution neural network. The structure, which is designed to predict the wavelet coefficients of high resolution image, consists of hidden layers and sub-pixel convolution layers. However, the wavelet method combines all the sub-bands to learn the image features without considering the differences between the sub-bands. For instance, the low-frequency sub-band reflects the main energy of the image, whereas the high-frequency sub-band focuses on the detailed information of the image in wavelet domain.

### 2.2. Brief Introduction of Efficient Convolutional Neural Networks

A series of existing methods has been proposed in recent years to enhance the deep neural network. Chollet presented the Xception [38], which mentions extreme inception and depthwise separable convolutions consisting of depthwise that convolute each channel independently and pointwise transform the depth of channels. Subsequently, ShufflNnet [39] utilizes channel shuffle to exchange the information of different channel groups. Howard et al. [40] proposed the third version of MoblileNet to reduce the redundant operations and parameters.In the first version, a framework was proposed based on depthwise separable convolution, which replaces the standard convolutions to reduce calculation. Subsequently, the second version noticed the linear bottlenecks and adopted linear activation instead of ReLU in low dimensional space. In addition, inverted residual blocks are used to enhance the generalization ability of the model. For the third version, SE block and h-swish activation was used. Han et al. [41] designed a ghost block to generate feature maps efficiently, which obtains more image information with less parameters. Ouahabi et al. [42] proposed an efficient network for medical image semantic segmentation. In their work, the dense connectivity, dilated convolutions, and factorized filters are organized into a new layer, which can improve accuracy and efficiency.

## 3. Proposed Approach

### 3.1. 2D Discrete Stationary Wavelet Transform

WFSAN is based on discrete stationary wavelet transform with haar function, which also named Db1 wavelet. The mother wavelet(wavelet function) of haar wavelet is ψ(x),and the father wavelet(scaling function) is ϕ(x), as shown by the following equation:

(1) $$\psi \left(x\right)=\left\{\begin{array}{cc} 1 & 0\leq x\leq 1/2\\ -1 & 1/2\leq x\leq 1\\ 0 & otherwise \end{array}\right.,\phi \left(x\right)=\left\{\begin{array}{cc} 1 & 0\leq x\leq 1\\ 0 & otherwise \end{array}\right.$$

The 2D discrete stationary wavelet transform can be regarded as performing 1D discrete wavelet transform in rows and columns. The decomposition and reconstruction of 1D-SWT can be described by discrete filters and sampling filters. In the decomposition, the high-pass filter is *H*, and the low-pass filter is *L*. i=1,2,3,…,N represents the level of wavelet decomposition.

Compared with discrete wavelet transform, SWT does not need the downsampling operator. The four sub-band coefficients, *A*, *H*, *V*, and *D*, represent the average, horizontal, vertical, and diagonal sub-band image, respectively. The subscript i represents the decomposition levels. For instance, $D_{1}$ represents the diagonal sub-band coefficients of one-level wavelet decomposition. Corresponding, the sub-band coefficients of level i+1 can be generated from coefficients of level *i* as follows:

(2) $$\begin{array}{cc} A_{i+1} & =L(L\left(A_{i}\right)),\\ H_{i+1} & =H(L\left(A_{i}\right)),\\ V_{i+1} & =L(H\left(A_{i}\right)),\\ D_{i+1} & =H(H\left(A_{i}\right)). \end{array}$$

**Figure 1:**
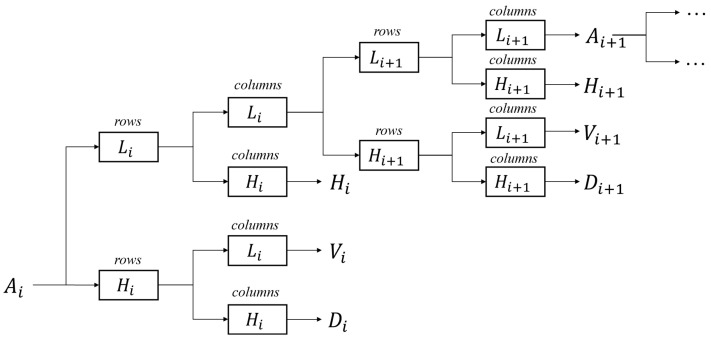
The diagram of two-dimension discrete stationary wavelet transform.

Figure 1 shows the 2D discrete stationary wavelet decomposition of *i* level. In one-level 2D-SWT, 2D signals are considered 1D signals among the rows. Thus, the coefficients are captured by performing 1D-SWT in rows and then in columns.

**Figure 2:**
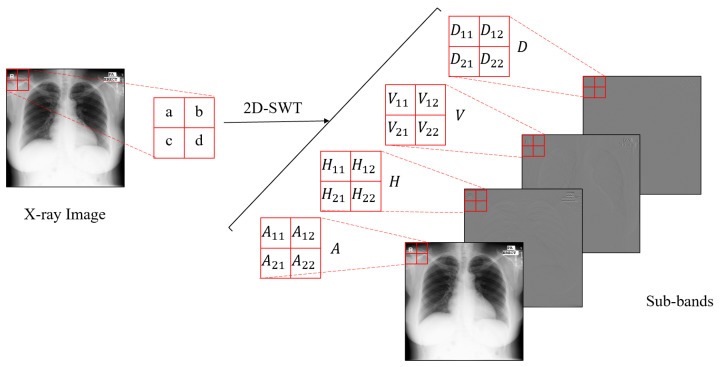
2D-SWT of a X-ray image using one-level.

Figure 2 a–d show four example pixels located in a 2×2 grid at the upper left corner of the original image. $A_{11},A_{12},A_{21},$ and $A_{22}$ can be seen as the linear combination of a, b, c, and d in Equation (3). It is similar in other sub-bands. We can obtain the sub-band coefficients of input image with 1-level 2D-DWT and predict the corresponding sub-band coefficients of the high resolution image.

With haar kernel in the 2D discrete stationary wavelet decomposition, the relationship between the pixel values and coefficients can be computed as follows:

(3) $$\left\{\begin{array}{ccc} A & = & \left[\begin{array}{cc} A_{11} & A_{12}\\ A_{21} & A_{22} \end{array}\right]=\left[\begin{array}{cc} \frac{1}{2}\left(a+b+c+d\right) & \frac{1}{2}\left(a+b+c+d\right)\\ \frac{1}{2}\left(a+b+c+d\right) & \frac{1}{2}\left(a+b+c+d\right) \end{array}\right]\\ H & = & \left[\begin{array}{cc} H_{11} & H_{12}\\ H_{21} & H_{22} \end{array}\right]=\left[\begin{array}{cc} \frac{1}{2}\left(a+b-c-d\right) & \frac{1}{2}\left(a+b+c+d\right)\\ \frac{1}{2}\left(-a-b+c+d\right) & \frac{1}{2}\left(-a-b+c+d\right) \end{array}\right]\\ V & = & \left[\begin{array}{cc} V_{11} & V_{12}\\ V_{21} & V_{22} \end{array}\right]=\left[\begin{array}{cc} \frac{1}{2}\left(a-b+c-d\right) & \frac{1}{2}\left(-a+b-c+d\right)\\ \frac{1}{2}\left(a-b+c-d\right) & \frac{1}{2}\left(-a+b-c+d\right) \end{array}\right]\\ D & = & \left[\begin{array}{cc} D_{11} & D_{12}\\ D_{21} & D_{22} \end{array}\right]=\left[\begin{array}{cc} \frac{1}{2}\left(a-b-c+d\right) & \frac{1}{2}\left(-a+b+c-d\right)\\ \frac{1}{2}\left(-a+b+c-d\right) & \frac{1}{2}\left(a-b-c+d\right) \end{array}\right] \end{array}\right.$$

**Figure 3:**
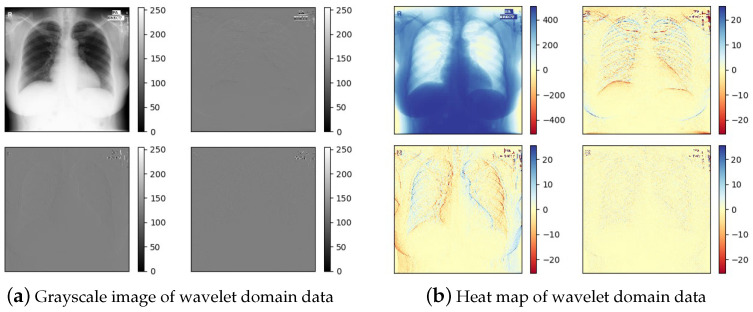
Grayscale image and heat map of the same transformed image in wavelet domain.

**Figure 4:**
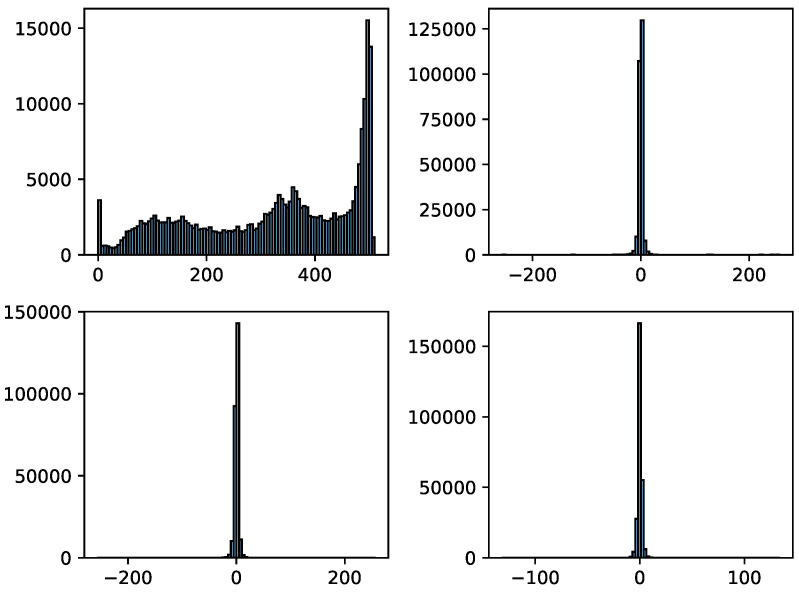
The statistic disturbutio of a two-dimensional discrete stationary wavelet transformed image.

The pixel values of the image and coefficients of the sub-bands from the corresponding image are represented by *a*, *b*, *c* and *d* and *A*, *H*, *V* and *D*. As shown in Figure 3b, a sample image is analyzed to indicate the range of coefficients, where *A* belongs to [0, 510], *H* belongs to [−255, 255], *V* belongs to [−255, 255], and *D* belongs to [−131, 133]. As displayed in MATLAB or the library matplotlib of the Python language, the grayscale image of wavelet domain data (a) is normalized to [0, 255]; it is easy to observe, but not at its authentic value. The range of pixel values in the original image is [0, 255]; thus, it can be computed by Equation (3), where the range of sub-band *A* is [0, 510] and that of *H*, *V* and *D* is [−255, 255]. Figure 4 shows that the approximate sub-band data are distributed in the interval of [0, 510], and the other sub-band data are almost approximately 0. The mean value for each sub-band is calculated to analyze the data characteristics. Concretely, according to Equation (3), it is clear that the sum of all elements in the *D* Matrix is 0; thus, its mean is 0. Similarly, the mean values of *H* and *V* are both 0. The experiment indicates that the mean value of *A* is 319.93, and the mean values of *H*, *V* and *D* are 0. Besides this, the standard deviations of *A*, *H*, *V* and *D* are 149.43, 12.55, 7.77, and 4.14, respectively. These features in stationary wavelet transform domain are considered in network design.

### 3.2. Network Architecture

**Figure 5:**
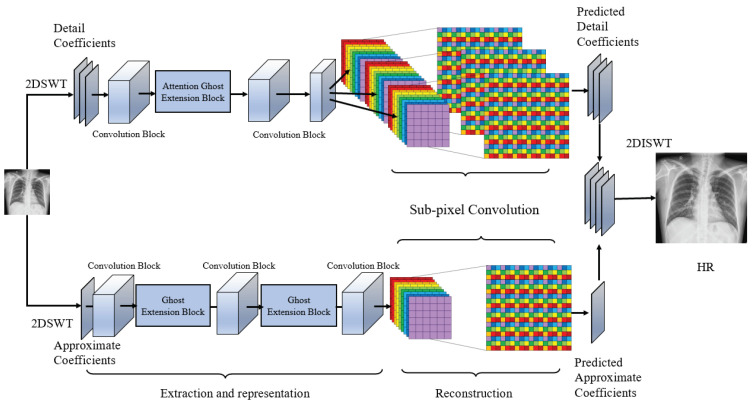
The network structure of WFSAN. The inputs are separated into approximate and detail sub-band coefficients of low-resolution image. The network output is the combination of approximate and detail sub-band coefficients of the predicting image.

We present a novel framework for medical imaging super-resolution, which considers the data features of wavelet domain. As illustrated in Figure 5, the WFSAN model can be decomposed into feature extraction, representation net, and reconstruction net. The part of extraction and representation is further divided into approximate and detail frequency sub-band extraction and representation. Different attention ghost extension blocks are designed to capture the features of each separated wavelet frequency sub-band individually. Subsequently, these features are used for reconstructing with sub-pixel convolution. The output of each sub-band is fused to generate the final image.

We represent the input image as $I_{LR}$. Approximate sub-band coefficients and detail sub-band coefficients of input low resolution image are $L_{CA}$ and $L_{CD}$, whereas $H_{CA}$ and $H_{CD}$ represent approximate sub-band coefficients and detail sub-band coefficients of the output high-resolution image $I_{HR}$. Moreover, $L_{CD}$ consists of three sub-bands, namely $L_{cV}$, $L_{cH}$, and $L_{cD}$, corresponding to vertical, horizontal, and diagonal information, respectively. $f_{s}$ indicates separating function, and $f_{c}$ is a combination function. $f_{swt}$ and $f_{iswt}$ indicate the discrete stationary wavelet transform and its inverse transform. In feature extraction, two block types are designed to extract the features from different sub-bands.

(4) $$\begin{array}{cc} L_{CA},L_{cH},L_{cV},L_{cD} & =f_{s}\left(f_{swt}\left(L_{LR}\right)\right);\\ L_{CD} & =f_{c}\left(L_{cH},L_{cV},L_{cD}\right);\\ F_{A} & =f_{A}\left(L_{CA}\right),F_{D}=f_{D}\left(L_{CD}\right), \end{array}$$

where $f_{A}\left(\cdot \right)$ and $f_{D}\left(\cdot \right)$ represent the low-frequency (approximate) and high-frequency (detail) feature extraction network, respectively, consisting of attention ghost extension blocks and standard convolutions. As the outputs of extraction operation, $F_{A}$ and $F_{D}$ are input into the reconstruction net to predict the coefficients, where *U* denotes the upsampling operation that consists of the sub-pixel convolution layer. The reconstruction net is designed to transform the fused features to residual wavelet coefficients. Ultimately, the predicted high-resolution image is generated by the following:

(5) $$I_{HR}^{{}^{\prime }}=f_{iswt}\left(U\left(F_{A}\right),U\left(F_{D}\right)\right)$$

The loss based on most common loss $l_{2}$ is adopted to predict the approximate and detail coefficients, which can be defined as follows:

(6) $$Loss=\frac{1}{2N}\sum_{n=1}^{N}\left(\|H_{CA},-U\left(f_{A}\left(L_{CA}\right)\right){\|}^{2}+\|H_{CD}-U\left(f_{D}\left(L_{CD}\right)\right){\|}^{2}\right).$$

Fundamentally, we aim to learn the differences between the sub-band coefficients of low-resolution image and high-resolution images. Under the sub-pixel layers, we combine these sub-bands to generate the final high-resolution image with inverse discrete stationary wavelet transform.

### 3.3. Wavelet Frequency Separation Feature Extraction

The wavelet frequency separation feature extraction networks for approximate and detail frequency are designed given the different characteristics of each sub-band. A component of the approximate frequency sub-band is trained to obtain abundant low-frequency information, and detail frequency sub-bands are learnt to enhance their ability in reserving the edge information. Most parts of the structure are similar to each other, as shown in Figure 5.

The approximate coefficient feature extraction block has five blocks, including two low-attention ghost extension block layers and three standard convolution layers. The input initially passes a 3×3×32 convolution layer with ReLU activation function. All activation functions in this block adopt ReLU to promote the convergence of the model, because all approximate coefficients are positive numbers. Then, it is fed to a low-attention ghost extension block to capture features individually. We utilize a convolutional layer with 1×1×32 filters to adjust the channel, considering that concatenation leads to computational burden and redundant information. More blocks are adopted in this sub-band than in the others, because most information is in the approximate frequency sub-band. The last convolution kernel is 3×3×32, followed by the sub-pixel layer with 3×3×1 filters.

For the detail coefficient feature extraction block, the input initially passes a convolutional layer with Tanh activation function. The kernel is 3×3×32. Then, it is fed to the attention ghost extension block to capture features independently. The channels are reduced by a convolutional layer with a 1×1×32 filter. Furthermore, few filters are used in this path due to its sparsity. Moreover, the sub-pixel layer with 3×3×3 filters is adopted to reconstruct three coefficient feature maps in the detail sub-band. Tanh activation function is selected, because not all detail coefficients are positive. Finally, these sub-bands are merged together to generate the high-resolution image prediction through the inverse wavelet transform.

### 3.4. Attention Ghost Extension Block

**Figure 6:**
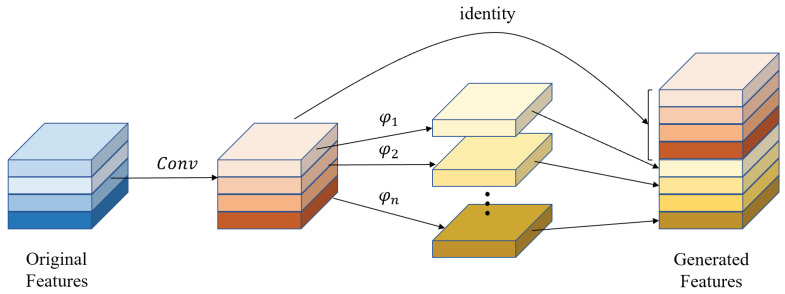
The ghost extension block.

Inspired by the ghost model [41] and convolutional block attention module [25], the attention ghost extension block is designed to generate feature maps efficiently. First, the ghost extension block is designed, as shown in Figure 6. The 3×3×32 kernel is used to form half the final feature maps *F*. Additionally, φ [41] represents a linear operation in the following Equation (7). In this block, 3×3 depthwise convolution replaces the original convolution to reduce the parameters further. Lastly, these features are contacted together with a descriptor $F_{c}$. In summary, the ghost extension produced can be formulated as follows:

(7) $$F^{{}^{\prime }}=F_{c}\left(F,\varphi \left(F\right)\right)$$

**Figure 7:**
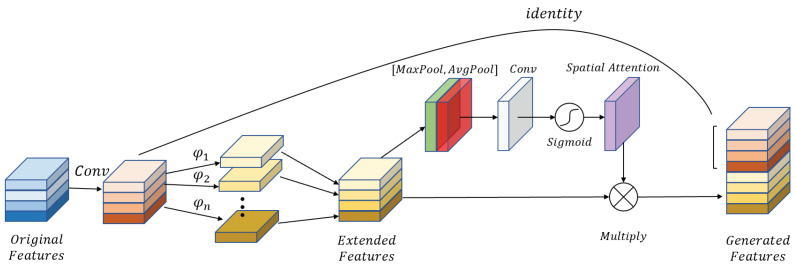
The attention ghost extension block with attention mechanisms in space.

Furthermore, to enhance the detail feature maps, the spatial attention mechanism is introduced in the attention ghost extension block, as shown in Figure 7. The same as the ghost extension block, half feature maps are generated with 3×3×32 convolution kernel. The final extension features are obtained from the ghost extension features, which are cascaded with spatial attention module. To capture more spatial features, the max-pooled features with salient information and average-pooled features with global information are exploited through channel max-pooling MaxPool(·) and average-pooling AvgPool(·). *f* represents the 3×3×1 convolution operation, which is used to merge $M_{avg}$ and $M_{max}$. The spatial attention feature map is normalized from the merged feature map with hard-sigmoid activation function σ. Eventually, the attention ghost extension feature maps are computed by element-wise multiplication ⊗ between the spatial attention feature map and ghost extension feature maps. Finally, two parts are contacted together. The overall process can be summarized as follows:

(8) $$\begin{array}{cc} F^{{}^{\prime \prime }} & =F_{c}\left(F,\varphi \left(F\right)⊗\sigma \left(f\left(AvgPool\left(\varphi \left(F\right)\right),MaxPool\left(\varphi \left(F\right)\right)\right)\right)\right)\\ \; & =F_{c}\left(F,\varphi \left(F\right)⊗\sigma \left(f\left(M_{avg},M_{max}\right)\right)\right) \end{array}$$

In Table 1, *N* represents the number of the channel, and H×W is the size of the input feature maps; *k* is the convolution kernel size, and *C* is the sum of filters; and *M* is the sum of the channels of input feature maps.

We compare the parameters and floating point operations(FLOPs) in each block. It indicates that the parameters and FLOPs of ghost extension block and attention ghost extension block are closed, but they are relatively smaller than those of the mini grid network.

## 4. Experimental Results

### 4.1. Data Set for Training and Testing

During the training phase, half a public data set, Shenzhen Hospital X-ray Set [43], with 662 X-ray images, and the Montgomery Set, with 138 images, were selected. During the testing phase, the remaining images were adopted. We cropped the rest of the images and resized them to 512×512 size, considering that the chest is only part of the Montgomery set image. Images were cropped to 48×48 pixel sub-images with 48 pixels overlapping for training. The batch size was set to 128. A total of 10% images were used for valid data set, and the remaining images were used for a test data set, which include normal and abnormal chest images of the two data sets. One channel information of these grayscale images is used in training and testing.

### 4.2. Quantitative Results

We compare the proposed WFSAN with three lightweight single image super resolution methods on two commonly used image quality metrics, namely PSNR and SSIM, as shown in Table 2. The best results are presented in red, and the second best results are presented in blue. Peak signal-to-noise ratio (PSNR) and structural similarity index (SSIM) are used to evaluate quantitative performance. Given two images, *I* and $I^{{}^{\prime }}$, which have the same size m×n, PSNR is defined as follows:

(9) $$\begin{array}{cc} \; & MSE=\frac{1}{mn}\sum_{i=1}^{m}\sum_{j=1}^{n}{\left[I\left(i,j\right)-I^{{}^{\prime }}\left(i,j\right)\right]}^{2};\\ \; & MAX_{I}=255;\\ \; & PSNR=10*log10(\frac{MAX_{I}^{2}}{MSE}), \end{array}$$

where $MAX_{I}$ represents the maximum possible pixel value, which is 255 here, because *I* and $I^{{}^{\prime }}$ are 8 bit images. PSNR is the most common and widely used objective measurement method to describe the image quality. The higher PSNR indicates better reconstruction image. Meanwhile, the SSIM can be defined as follows:

(10) $$SSIM=\frac{\left(2\mu_{I}\mu_{I^{{}^{\prime }}}+c_{1}\right)\left(2\sigma_{II^{{}^{\prime }}}+c_{2}\right)}{\left(\mu_{I}^{2}+\mu_{I^{{}^{\prime }}}^{2}+c_{1}\right)\left(\sigma_{I}^{2}+\sigma_{I^{{}^{\prime }}}^{2}+c_{2}\right)},$$

where $\mu_{I}$ and $\mu_{I^{{}^{\prime }}}$ represent the mean of image blocks *I* and $I^{{}^{\prime }}$; $\sigma_{I}^{2}$ and $\sigma_{I^{{}^{\prime }}}^{2}$ are their variances, respectively; $\sigma_{II^{{}^{\prime }}}$ is a covariance; and $c_{1}$ and $c_{2}$ are constants to maintain stability. The range of SSIM is from 0 to 1. The value is 1 when the two images are exactly the same. Three different methods are compared with our proposed method, and the bicubic algorithm is used as baseline reference.

The methods compared are SRCNN [16], FMISR [27], and WMSR [37], among which FMISR and WMSR have achieved lightweight medical imaging with super resolution and state-of-the-art performance in the last two years. To ensure the accuracy of empirical results, we have calculated the average values of PSNR and SSIM for all images from above image datasets in Table 2. Concretely, these results are obtained from 130 images of ChinaSet-Normal Dataset, 134 images of ChinaSet-Abnormal Dataset, 32 images of MontgomerySet-Normal Dataset, and 23 images of MontgomerySet-Normal Dataset, respectively.

In Table 2, taking advantage of wavelet WMSR and WFSAN can achieve a higher score in SSIM on all datasets. Our proposed method achieves competitive performance but uses fewer parameters. In particular, the proposed WFSAN advances WMSR [37] with the improvement margins of 0.48, 0.18, 0.65, and 0.62 dB on scale factor of ×2. In addition, our proposed approach obtains the top two results in SSIM only, except of abnormal ChinaSet chest imaging. This finding indicates that our wavelet frequency separation structure with attention ghost extension block not only reduces the parameters but also slightly improves quality. In addition, FMISR performs better on the ChinaSet dataset, and WMSR performs better on the Montgomery dataset. Our proposed method has competitive results on all datasets, owing to the generalization ability of the model.

**Figure 8:**
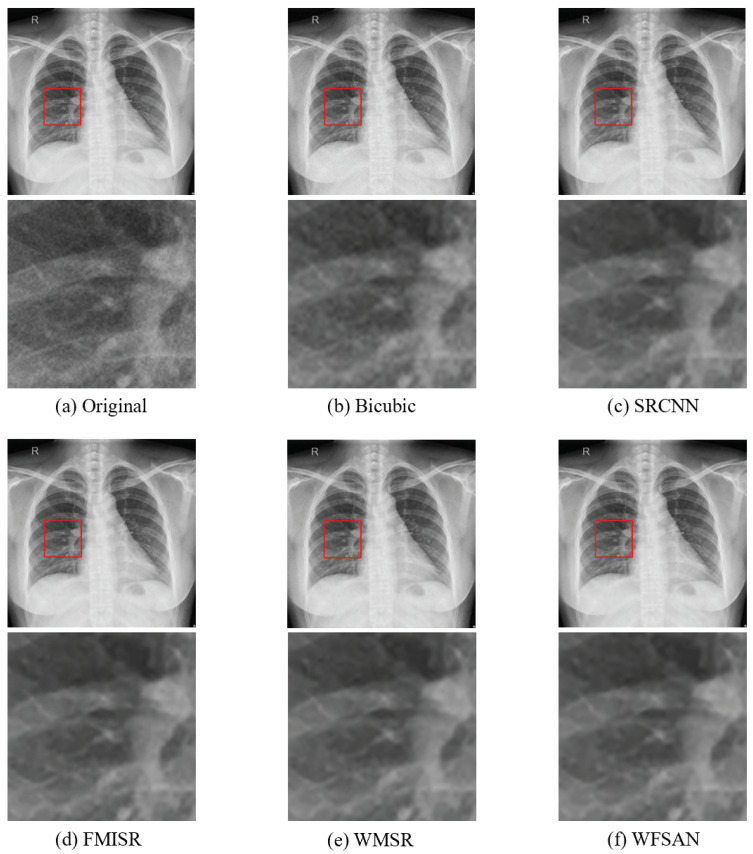
Visual comparison of different models in ChinaSet normal chest dataset, (**a**) Original(HR) image, (**b**) Bicubic, (**c**) SRCNN, (**d**) FMISR, (**e**) WMSR, (**f**) WFSAN.

**Figure 9:**
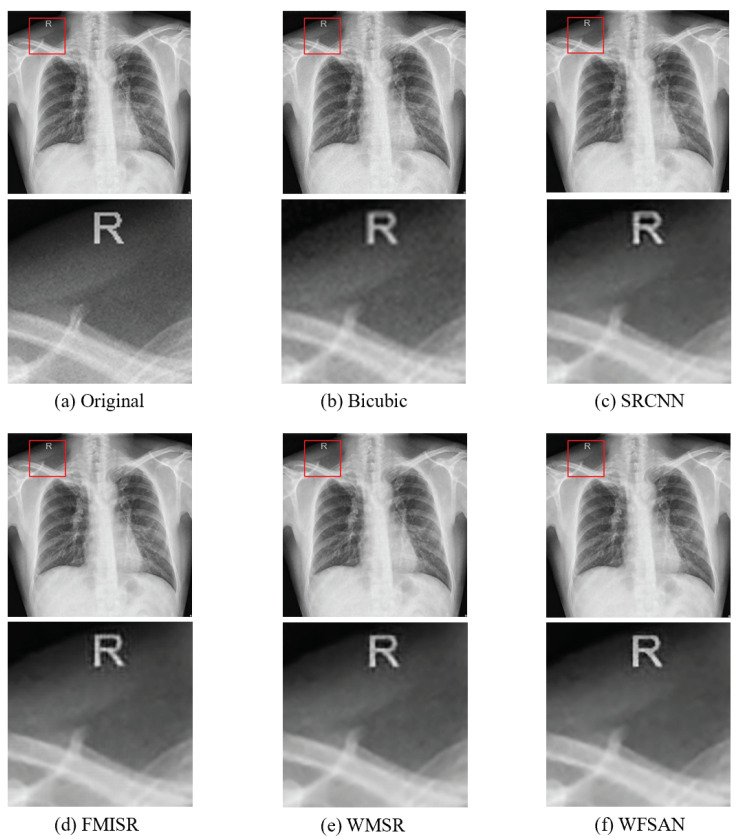
Visual comparison of different models in ChinaSet abnormal chest dataset, (**a**) Original(HR) image, (**b**) Bicubic, (**c**) SRCNN, (**d**) FMISR, (**e**) WMSR, (**f**) WFSAN.

**Figure 10:**
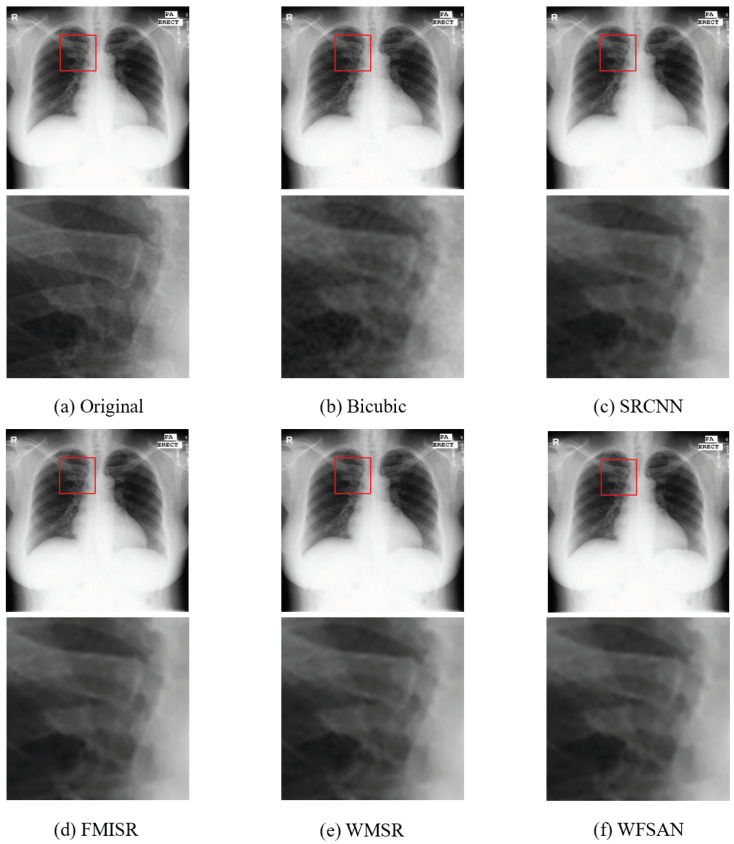
Visual comparison of different models in MontgomerySet normal chest dataset, (**a**) Original(HR) image, (**b**) Bicubic, (**c**) SRCNN, (**d**) FMISR, (**e**) WMSR, (**f**) WFSAN.

**Figure 11:**
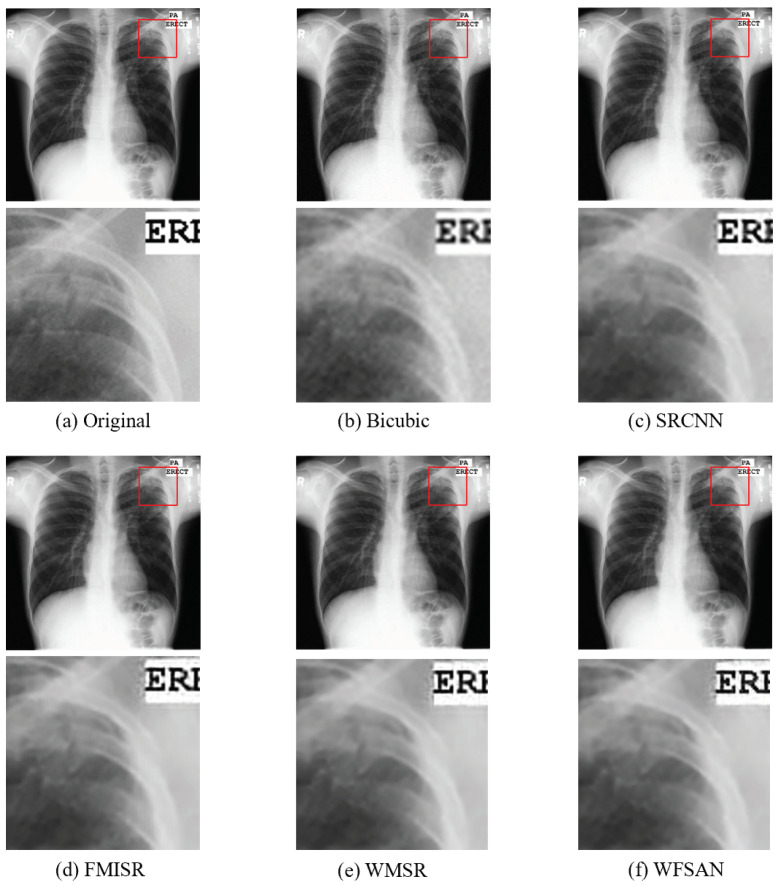
Visual comparison of different models in MontgomerySet normal chest dataset, (**a**) Original(HR) image, (**b**) Bicubic, (**c**) SRCNN, (**d**) FMISR, (**e**) WMSR, (**f**) WFSAN.

The visual comparisons of different methods are presented in Figure 8, Figure 9, Figure 10 and Figure 11. From these figures, it can be seen that the reconstructed image is evidently the closest to the original image by using our WFSAN model. Particularly, the letters in Figure 9 and Figure 11 are more coherent and cleaner than the other methods.

Furthermore, we have tested the methods on the training machine. Table 3 presents the execution time for each method on this computer. Our proposed approach has less parameters than the other methods. The proposed method and WMSR are slower than the FMISR, because the tensorflow framework does not support the wavelet transform directly. In addition, the sub-pixel convolution layer has no optimization in tensorflow, compared with the standard convolution layer. The number of sub-pixel convolution layers is four times that of the FMISR and WMSR. This condition influences the time to apply the high-resolution image. Ultimately, we can observe that the proposed approach is faster than the SRCNN in the tensorflow framework.

**Figure 12:**
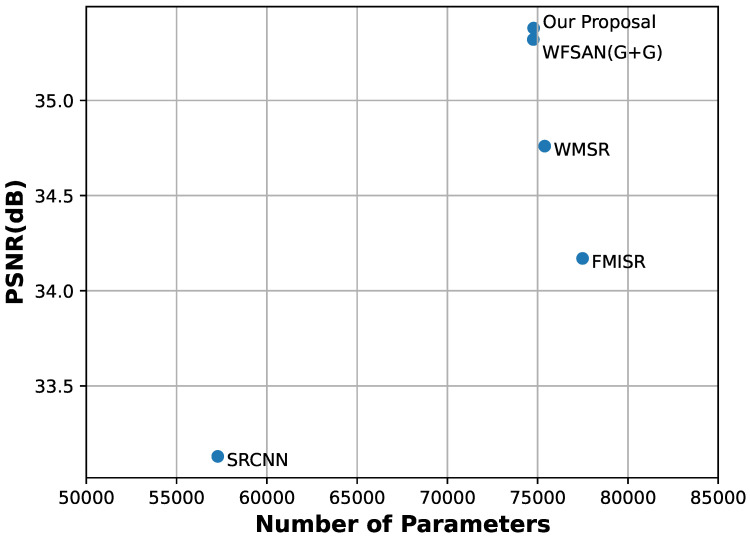
PSNR(dB) versus number of parameters for different methods on MontgomerySet abnormal data set.

Figure 12 indicates that SRCNN has the lowest PSNR with the least parameters. Although the parameters of the proposed approach are few, we still obtained competitive results. The WFSAN(G+S), which we adopt finally, has favorable performance in PSNR with very slight increase in the parameters.

### 4.3. Implementation Details

We use tensorflow framework to implement our proposed approach with Python3.7 interaction interface. The hardware devices include 32GB size of memory, NVIDIA GeForce GTX 1080Ti GPU, and Intel(R) Core(TM) i7-6850K CPU@3.60GHz. Meanwhile, the experimental platform includes Matlab2018a, Anaconda3, CUDA Toolkit v10.0, and Tensorflow2.0.

We train our model in ×2, ×3, and ×4, because our proposed method can only process a single-scale factor. Meanwhile, we use the $l_{2}$-based loss function Formula (6) instead of the $l_{2}$ loss. These several training techniques are used during the training process. We learn the independent maps to reconstruct the separated wavelet frequency information instead of learning the transform from a complete low-resolution image to restore the super-resolution image directly. Detail sub-band learning is used to increase the sparsity and reduce the complexity. The gradients are clipped to 0.001 by norm clipping option in the training. We select the Adam optimizer to update Θ and *b*. The initial learning rate is 0.001 and decreases through a cosine decay method (Algorithm 1).
**Algorithm 1** Cosine decay function1:lr=initial_leaning_rate, α=0.0001, decay_epoch=30;2:**while** epoch<max_epoch **do**3:     **if** Mod(epoch,decay_epoch)==0 **then**4:            lr=lr*0.1;5:     **else**6:            lr=(1−α)*(0.5*(1+cos(π*epoch/decay_epoch)))+α;7:     **end if**8:**end while**

The decay_epoch is set to 100, and the α is set to 0.0001 in the training procedure. The training procedure takes about 10 h with GPU. Our network is fully converged in 100 epochs, and (Θ,b) is used for testing. We train the model in 100 epochs after the pretraining, because large-scale datasets are difficult to converge. For fair comparison, the entire learning-based methods are trained and tested on the same proposed datasets.

Two combinations of ghost module extension (GBE) block and spatial attention ghost module extension (SAGBE) are tested to decide the structure of attention ghost extension block, as shown in Table 4. The first combination, called WFSAN(G+G), utilizes the GBE in the approximate frequency sub-band and detail frequency sub-band. Meanwhile, WFSAN(G+S) utilizes the GBE in approximate frequency sub-band and SAGBE in detail frequency sub-band. The result implies that WFSAN(G+S) performs better in PSNR (dB) and SSIM in general. Therefore, we select the combination of WFSAN(G+S).

### 4.4. Discussion

As mentioned above, it is clear that the wavelet-based super resolution methods [34,37] can obtain high resolution images effectively. However, their methods tend to mix up the approximate and the detail information in the process of prediction. This will not take full advantages of the global and local information of the X-ray image. Therefore, to obtain more information from the input images, we design a lightweight wavelet frequency separation attention network in our work. Experimental results of the proposed work demonstrate the effectiveness of our lightweight super resolution method. However, due to the factor that the lightweight model does not have sufficient capacity, the scale of wavelet decomposition is selected as one level. On the other hand, to extract more features, we design a spatial attention mechanism in our work. Unlike GhostNet, the attention ghost extension block with spatial attention mechanism can achieve more detail information than a channel attention mechanism. This can be attributed to two factors. One is that the scale of average-pooling based channel attention will be close to zero. The other is that the spatial attention mechanism can pay attention to more local information.

As a result, according to the comparison of Section 4.2, we can see that the proposed spatial attention mechanism has better performance than works FMISR [27] and WMSR [37] in terms of PSNR and SSIM. However, the reconstructed X-ray image is too smooth, to some extent, in our experiments. To address this issue, we will combine the optimization and deep learning methods in our next work.

## 5. Conclusions

We propose an effective wavelet frequency separation attention network single-image super-resolution method WFSAN for medical imaging reconstruction, which utilizes features in approximate frequency sub-band coefficients and enhances features in detail frequency sub-band coefficients in the wavelet domain. The use of learning detail coefficients, which are sparse, independently promotes the convergence. Ghost extension block and attention ghost extension block are designed to reduce the parameters and improve the information for each sub-band. In addition, these sub-band coefficients are combined to reconstruct all the coefficients. Eventually, we generate the high-resolution image through the inverse stationary wavelet transform.

The proposed approach is advantageous in memory with competitive quality results compared with other lightweight deep learning methods. In the future, we will analyze other wavelets of the wavelet family. Furthermore, statistical methods are considered to analyze the numerical information of high-resolution image and low-resolution image in the wavelet domain to provide a better normalization method. Detail sub-band coefficients should be generated from low-resolution image directly. Moreover, we have attempted to use the complex wavelet transform, which did not provide favorable results, because we cannot train the data in the complex domain directly. Therefore, we will focus on the super-resolution in the complex wavelet domain.

## Figures and Tables

**Table 1:** Comparison of mini grid network, ghost extension block, and attention ghost extension block.

Method	Parameters	FLOPs
Mini Grid Network	$\left(N\times k^{2}+1\right)\times C+\left(C\times k^{2}+1\right)\times M$	$2k^{2}\times C\times H\times W\times \left(N+M\right)$
Ghost Extension Block	$\left(N\times k^{2}+1\right)\times C+C\times k^{2}$	$2k^{2}\times C\times H\times W\times \left(N+1\right)$
Attention Ghost Extension Block	$\left(N\times k^{2}+1\right)\times C+C\times k^{2}+2k^{2}$	$2k^{2}\times C\times H\times W\times \left(N+1\right)+4k^{2}\times H\times W$

**Table 2:** Quantitative evaluation results of the different methods with PSNR(dB) and SSIM.

Data Set	Scale	Bicubic		SRCNN [16]		FMISR [27]		WMSR [37]		Our Proposal	
		PSNR	SSIM	PSNR	SSIM	PSNR	SSIM	PSNR	SSIM	PSNR	SSIM
ChinaSet-Normal	×2	32.83	0.8675	34.61	0.8905	35.05	0.8923	34.95	0.8949	35.43	0.8952
	×3	31.92	0.8450	32.57	0.8626	33.71	0.8681	32.79	0.8697	33.08	0.8700
	×4	29.91	0.8259	30.42	0.8327	31.39	0.8452	30.82	0.8451	31.12	0.8457
ChinaSet-Abnormal	×2	33.30	0.8445	34.02	0.8577	34.23	0.8584	34.26	0.8598	34.44	0.8608
	×3	32.29	0.8118	32.65	0.8232	33.10	0.8275	32.93	0.8294	32.97	0.8286
	×4	30.94	0.7869	31.22	0.7958	31.68	0.8020	31.46	0.8021	31.58	0.8018
MontgomerySet-Normal	×2	30.96	0.8974	32.83	0.9305	33.97	0.9354	34.66	0.9400	35.31	0.9383
	×3	29.14	0.8842	30.03	0.9085	31.55	0.9162	31.91	0.9184	31.60	0.9179
	×4	27.87	0.8724	28.44	0.8907	29.22	0.8967	29.32	0.8969	29.78	0.8990
MontgomerySet-Abnormal	×2	31.49	0.8940	33.13	0.9240	34.17	0.9284	34.76	0.9327	35.38	0.9323
	×3	29.64	0.8785	30.45	0.9009	31.89	0.9077	32.21	0.9097	31.97	0.9091
	×4	28.55	0.8656	29.04	0.8822	29.77	0.8894	29.93	0.8883	30.38	0.8906

**Table 3:** Computational time of different methods.

Execution Time for Different Method in Scale 4				
Method	SRCNN	FMISR	WMSR	Our Proposal
SR-Time/s	0.7557	0.2278	0.4156	0.7275

**Table 4:** Testing different combinations.

The Quantitative Results for Different Combinations		
Combination	WFSAN(G+G)	WFSAN(G+S)
Dataset	PSNR/SSIM	PSNR/SSIM
ChinaSet-Normal	35.23/0.8951	35.43/0.8952
ChinaSet-Abnormal	34.38/0.8602	34.44/0.8608
MontgomerySet-Normal	35.34/0.9379	35.31/0.9383
MontgomerySet-Abnormal	35.42/0.9307	35.38/0.9323

## Data Availability

Some or all data used during the study are available online in accordance with funder data retention polices (https://www.ncbi.nlm.nih.gov/pmc/articles/PMC4256233/#__sec2title).
